# Characteristics of SP600125 Induced Tetraploid Cells in Comparison With Diploid and Tetraploid Cells of Fish

**DOI:** 10.3389/fgene.2021.781007

**Published:** 2021-12-06

**Authors:** Yunpeng Fan, Guangjing Zhang, Kaiyue Zhao, Wen Fu, Shujuan Chen, Jinhui Liu, Wenbin Liu, Liangyue Peng, Li Ren, Shaojun Liu, Yamei Xiao

**Affiliations:** ^1^ State Key Laboratory of Developmental Biology of Freshwater Fish, Hunan Normal University, Changsha, China; ^2^ College of Life Sciences, Hunan Normal University, Changsha, China

**Keywords:** SP600125, tetraploid, mitochondrion, cell cycle, RNA-seq

## Abstract

In our previous research, SP600125 (Anthrapyrazolone) was used to induce autotetraploid of crucian carp cells (SP4N cells), and tetraploid fry was generated from the SP4N cells by somatic cell nuclear transfer technique. However, it is still unclear about biological characteristics of the SP4N cells. In this article, the cytological characteristic and gene expression profiles of the SP4N cells are investigated in comparison with the crucian carp cells (2N cells) and the tetraploid crucian carp cells (CC4N cells). The SP4N cells have tetraploid characteristics in terms of morphology and DNA ploidy levels, and their chromosome behavior is stable during the cell proliferation. The migration ability and the mtDNA copy number of SP4N cells are both lower than those in the CC4N cells and the 2N cells, but there exist giant mitochondria in the SP4N cells. The similar expression trends in the cell cycle regulation genes of the SP4N cells and 2N cells, while the corresponding expression profiles are clearly different between the SP4N cells and the CC4N cells. Moreover, the significant difference genes are associated with energy metabolism pathways among the SP4N cells, 2N cells and CC4N cells. These results can provide deeper understanding of SP600125 induction, as well as finding applications in polyploidization breeding of fish species.

## Introduction

Small compound SP600125 (Anthrapyrazolone), known as a c-Jun N-terminal kinase (JNK) inhibitor, is often used in research on JNK signaling pathway (Bennett et al., 2001). SP600125 can also affect cell apoptosis, progression of cancer, pathological response, and maintaining pluripotency of stem cells (Nakaya et al., 2009; Kim et al., 2010; Kook et al., 2013; Yao et al., 2014). Our previous study found that SP600125 could induce polyploidization in diploid crucian carp cells, and generated an autotetraploid cell line (named SP4N cells) (Zhou et al., 2016). Moreover, combining somatic cell nuclear transfer technique, autotetraploid fish was successfully generated from the SP4N cells (Zhou et al., 2016). However, little is known about the biological characteristics of the SP4N cells.

Polyploid is rare phenomenon in animals (Comai 2005; Blomme et al., 2006; Schoenfelder and Fox 2015). Tetraploid crucian carp is hybrids of *Carassius auratus red* var. and *Cyprinus carpio L.*, which is regarded as the first natural case of an allotetraploid vertebrate animal with reproductive fertility and stable genetic characters (Liu et al., 2001; Liu et al., 2016). In this research, cytological and transcriptome analysis will be conducted for the SP4N cells, the diploid cells from the caudal fin of *C. auratus* (2N cells), and the tetraploid cells from the caudal fin of tetraploid crucian carp (CC4N cells). Particularly, we attempt to answer the following question: Compared with the 2N cells and the CC4N cells, what are the cytological characteristics and the gene expression profiles of SP4N cells? Clearly, such a answer may provide more valuable knowledge for polyploid breeding of fish species.

## Materials and Methods

### Cell Culture

The SP600125-induced tetraploid (SP4N) cells were come from State Key Laboratory of Developmental Biology of Freshwater Fish, Hunan Normal University (Zhou et al., 2016). Diploid crucian carp fibroblast (2N) cells were obtained from the caudal fin of *C. auratus red* var., and tetraploid fish (CC4N) cells were derived from the caudal fin of a tetraploid hybrid of *C. auratus red* var. (♀) × *C. carpio L.* (♂).

Cells were cultured in Dulbecco’s Modified Eagle’s Medium (DMEM, Gibco, Life Technologies, CA, United States), supplemented with 100 U/ml penicillin, 100 μg/ml streptomycin (Invitrogen, Carlsbad, CA, United States), 10% fetal bovine serum (FBS, Invitrogen, Carlsbad, CA, United States), 0.1% 2-mercaptoethanol (2-ME, Invitrogen, Carlsbad, CA, United States), 1 mM sodium pyruvate (Invitrogen, Carlsbad, CA, United States), and 1 mM nonessential amino acids (Invitrogen, Carlsbad, CA, United States). And all of the cells used were from the 16th to 18th passage.

### Karyotyping

Confluent cells were treated with 0.1 μg/ml colchicine for 2–4 h before chromosome preparation. The cells were digested into single-cell by 0.25% trypsin (Invitrogen, Carlsbad, CA, United States). After hypotonic treatment with 0.075 mol/L KCl for 30–60 min, the cells were fixed twice with cold Carnoy’s fixative (methanol: glacial acetic acid = 3:1, v/v) for 20 min each time. Then, the cells were dropped onto the cold slides and stained with 5% Giemsa solution (Solarbio Inc. United States) for 15 min. More than 30 metaphases were examined for each cell line.

### Flow Cytometer Analysis

Analysis of DNA ploidy levels and nuclear C values were performed with a flow cytometer (Sysmex-partec, Germany). Cells were digested into single-cell suspension by 0.25% trypsin. After being filtered through a 40 μm cell strainer, the cells were incubated with 2 μg/ml Hoechst 33342 (Invitrogen) and 50 μM Verapamil (Sigma) for about 15 min.

### Immunofluorescence

After fixed in 4% paraformaldehyde for 30 min, cell samples were treated with 0.3% Triton X-100 for 3 min and blocked for 1 h with 0.2% bovine serum albumin (Calbiochem, San Diego, CA, United States). Anti-*α*-tubulin antibody (dilution ratio was 1:100; GeneTex, Inc. North America) was used as the primary antibodies. Anti-mouse IgG fluorescent secondary antibody (dilution ratio was 1:200) was purchased from Abways Biotechnology Co., Ltd. (Shanghai, China). DNA and mitochondria were stained with Hoechst 33342 (Invitrogen) and MitoTracker Green FM (Invitrogen), respectively.

### Observation by Scanning Electron Microscopy

Cells were fixed in 2.5% glutaraldehyde for 2 h, and then treated with 1% OsO4 for 2 h. After dehydration through an ethanol gradient, the specimens were embedded in Epon812 resin (TAAB, United States). The sections were double-stained with uranyl acetate and lead citrate, and were examined by an electron microscope (JEM-1230, JEOL, Tokyo, Japan).

### Cell Migration Test

Wound healing assay was used to examine the cell migration capacity. The wound was created perpendicular to the marking on 60-mm dish, then, washed with PBS until no cells at the scratch. Upon the pre-experiment basis, the sampling time point was 24 h. Images acquired were analyzed quantitatively by ImageJ software. For each sample, experiments were repeated at least 3 times.

### Analysis of ATP Content and mtDNA Copy Number

Cells were digested by 0.25% trypsin into a single-cell solution, washed 3 times with sterile physiological saline. After centrifugation, 500 μL ddH_2_O was added and homogenate was broken in a hot bath (95°C). Cellular ATP content was assessed using an ATP Assay Kit (Nanjing Jiancheng Bioengineering Institute, China) according to the manufacturer’s instructions. For each sample, experiments were repeated at least 3 times.

The copy number of mitochondrial DNA was determined by real-time fluorescence quantitative PCR (qPCR). The mtDNAs was extracted with DNA extraction kit (D3396-01, Omega). The *enc1* gene (nuclear single-copy gene) and *cytb* gene (mitochondrial gene) were selected for qPCR test. According to the multiple of *cytb* relative to *enc1* (Li et al., 2007), the number of mtDNA copies in a single cell was converted according to the number of template genomes. For each sample, experiments were repeated at least 3 times.

### Obtaining Transcriptome Data

For this research, we obtained mRNA sequencing (seq) data of *in vitro* diploid *C. auratus* red var. and tetraploid *C. auratus* red var. (♀) × *C. carpio L.* cultured cells from the NCBI SRA database (SRR7640867, SRR7640866, SRR7640869, and SRR7640868) (Ren et al., 2020). Next, we submitted the mRNA-seq data of SP600125-induced tetraploid cells to the NCBI SRA (SRR9964682 and SRR9964683).

### qRT-PCR Analysis

Total RNA was extracted using RNAiso Plus reagent (Takara, Bio. Beijing). The qRT-PCR was conducted as described in our previous research (Mo et al., 2019; Ren et al., 2020). Primers were designed using Primer Premier 5.0 software (Supplementary Table S2), and used to detect expression with the following amplification conditions: 50°C for 2 min and 95°C for 10 min, followed by 40 cycles at 95°C for 15 s and 60°C for 1 min. The average threshold cycle (Ct) was calculated for each sample using the 2−ΔΔCt method (Livak and Schmittgen, 2001). The housekeeping gene *β-actin* was used as the reference gene. For each sample, qRT-PCR analysis was conducted three times.

## Results

### Cytological Observation of SP4N Cells Comparison With Diploid and Tetraploid Fish Cells

**FIGURE 1:**
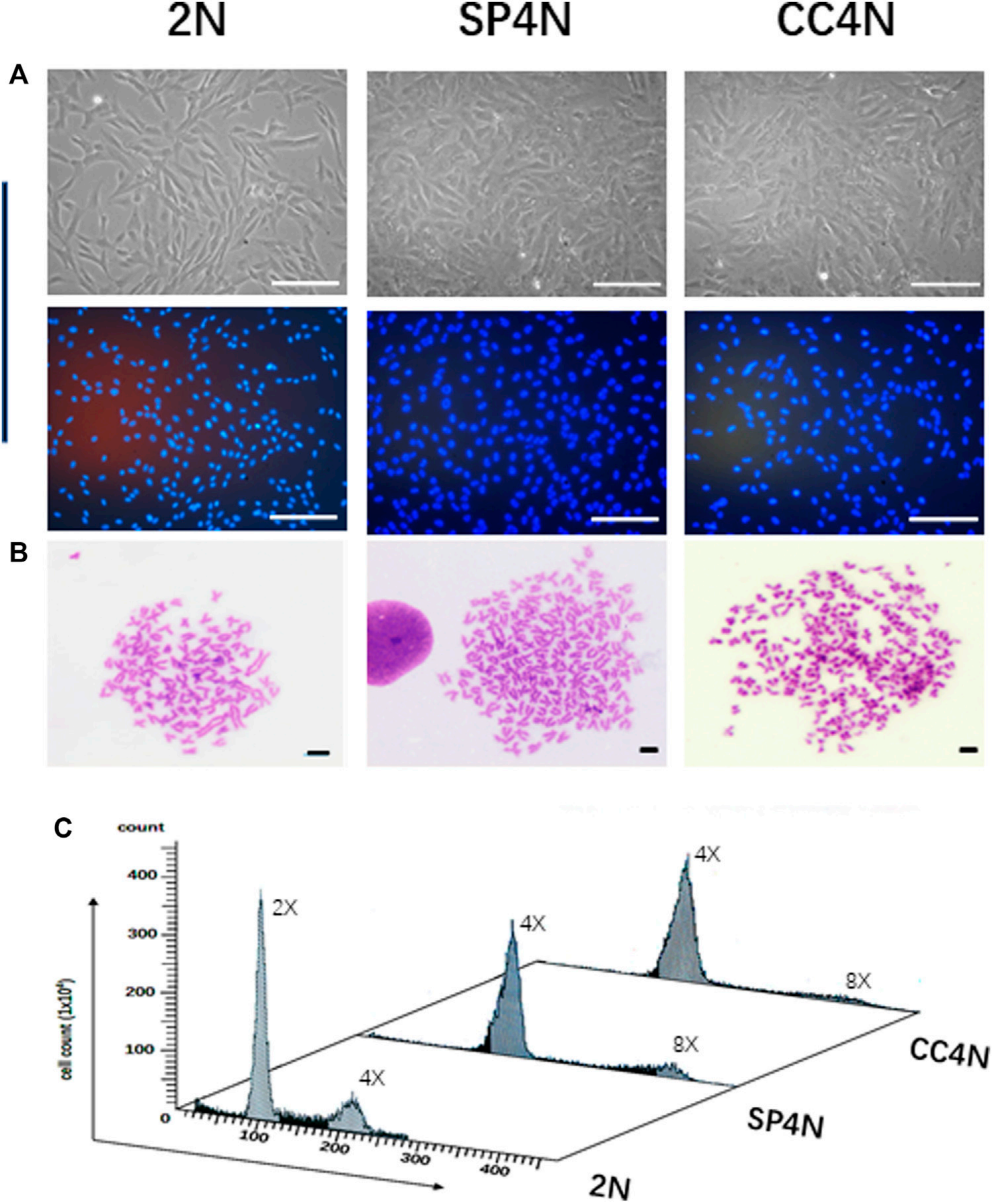
Karyotypes and DNA ploidy levels of the SP600125-induced tetraploid cells (SP4N cells), tetraploid cells from *C. auratus* × *C. carpio L.* (CC4N cells) and diploid fish cells (2N cells). **(A)** Morphological observation of the 3 cell lines (The cells in the second row were stained with Hoechst 33342), where all the scale bars represent 200 µm. **(B)** Karyotypes of the 3 cell lines, where all the scale bars represent 20 µm. More than 30 metaphases were examined per-sample. **(C)** DNA ploidy levels of the 3 cell lines were analyzed by flow cytometry. For each sample, experiments were repeated at least 3 times. Lanes from 1 to 3 showed the results for the 2N cells, SP4N cells and CC4N cells, respectively.

As shown in Figure 1, the SP4N cells and CC4N cells were coincident in size, where the long diameters were 103.5 ± 20.0 µm, and the short diameters were 45.5 ± 12.5 µm). In contrast, the long and short diameters of the 2N cells were 100.0 ± 12.0µm and 25.5 ± 8.5µm, respectively. The nuclei diameters of the SP4N cells and CC4N cells were about 42.5 ± 8.0 µm, while 22.5 ± 5.0 µm in that of the 2N cells (Figure 1A). In the SP4N cells and CC4N cells, there were about 70% of metaphases with 200 chromosomes and others were between 187 and 198. And the majority of the 2N cells (90%) contained 100 chromosomes, while for the other 10% of them, the number of chromosomes was between 93 and 98 (Figure 1B). Moreover, the relative DNA contents (C value) were about 4C in the SP4N cells and CC4N cells, while that in the 2N cells was 2C (Figure 1C). Flow cytometry analysis further demonstrated that there existed diploid (2x, 100) and tetraploid (4x, 200) peaks in the 2N cells, while the tetraploid (4x, 200) and octoploid (8x, 400) peaks display in the SP4N and CC4N cells (Figure 1C).

**FIGURE 2:**
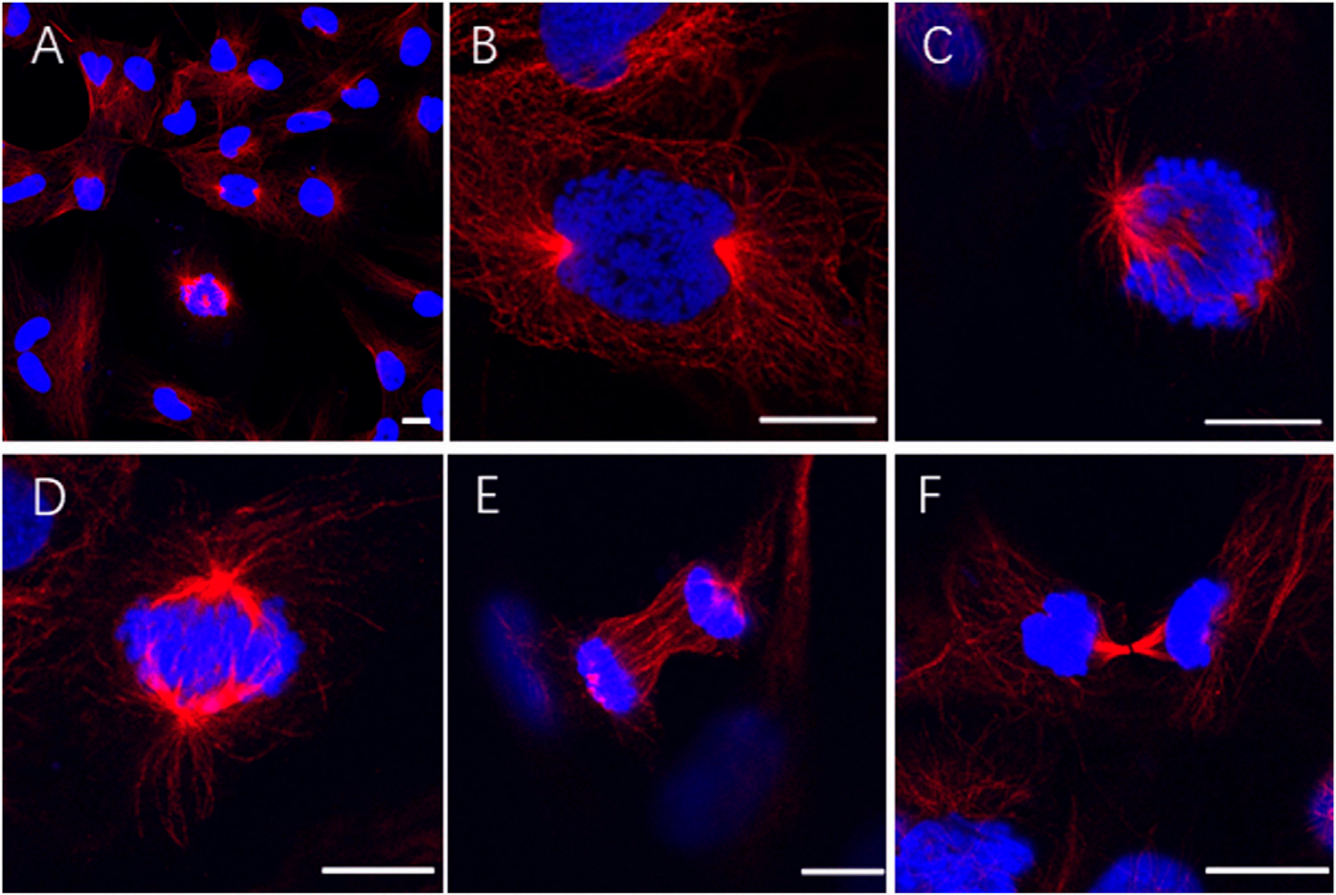
Immunofluorescence observation of chromosome behaviors in the SP600125-induced tetraploid (SP4N) cells. **(A)** presents 21 passages of the SP4N cells. **(B–F)** present the SP4N cells in prophase **(B)**, pro-metaphase **(C)**, metaphase **(D)**, anaphase **(E)** and telophase **(F)** of the mitosis, respectively. *α*-Tubulin (red) was detected by immunostaining, and DNA (blue) was stained with Hoechst 33342. All the scale bars represent 10 µm. Cell migration ability and observed mitochondria in three types of cells.

By immunofluorescence staining, normal chromosome behaviors were observed in the mitosis of SP4N cells (Figures 2A–F). The growth of SP4N cells was stable during subsequent subculture such that more than 38 passages were expanded (Supplementary Figure S1).

**FIGURE 3:**
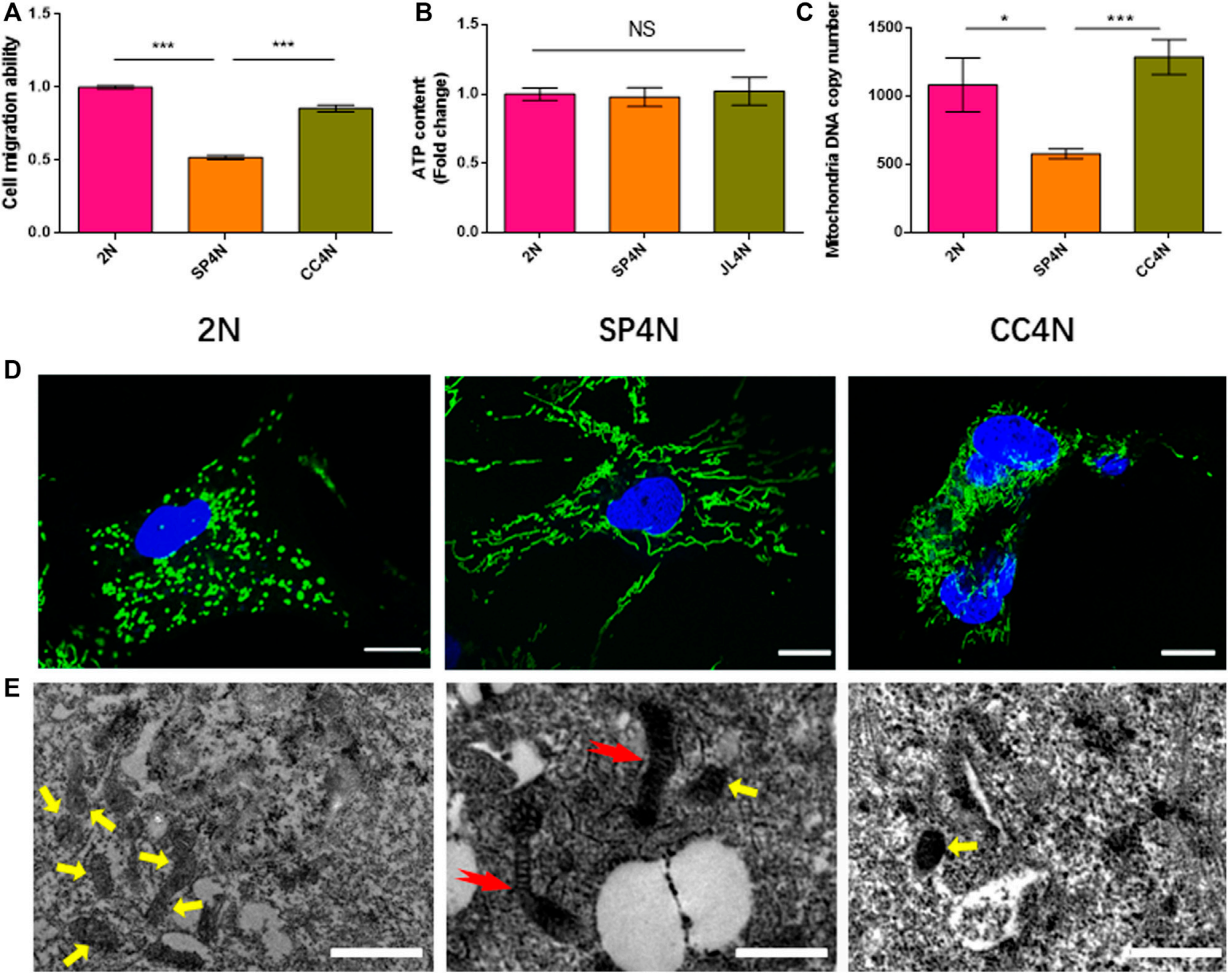
Cell migration ability and observed mitochondria in the SP600125-induced tetraploid cells (SP4N cells), tetraploid cells from *C. auratus* × *C. carpio L.* (CC4N cells) and diploid fish cells (2N cells). **(A–C**) present the cell migration capacity, the ATP contents and the mtDNA copy number, respectively. “NS” stands for no significant difference, *p ≤ 0.05, **p ≤ 0.01 and ***p ≤ 0.001 represent different significance levels of *t*-test, respectively. **(D)** presents the observed mitochondria by laser confocal microscope. Mitochondria (green) and DNA (blue) were stained with MitoTracker Green FM and Hoechst 33342, respectively. The scale bars represent 10 µm. **(E)** shown the observed mitochondria by transmission electron microscope, where the scale bars represent 1 µm. The yellow arrows point to small oval mitochondria, and red arrows point to giant mitochondria. DE analysis between SP4N cells and other two cells using mRNA-Seq.

The results of wound healing assay indicated that the migration ability of SP4N cells was the weakest among the three fish cell lines, and that of the 2N cells was stronger than that of the CC4N cells (Figure 3A). There was no significant difference in the levels of ATP contents under unit protein concentration among the three types of cells (Figure 3B). However, the mtDNA copy number of SP4N cells was significantly lower than those of the 2N and CC4N cells (Figure 3C). The 2N cells had more mitochondria than the SP4N and CC4N cells, but giant mitochondria were observed in the SP4N cells (Figures 3D,E).

**FIGURE 4:**
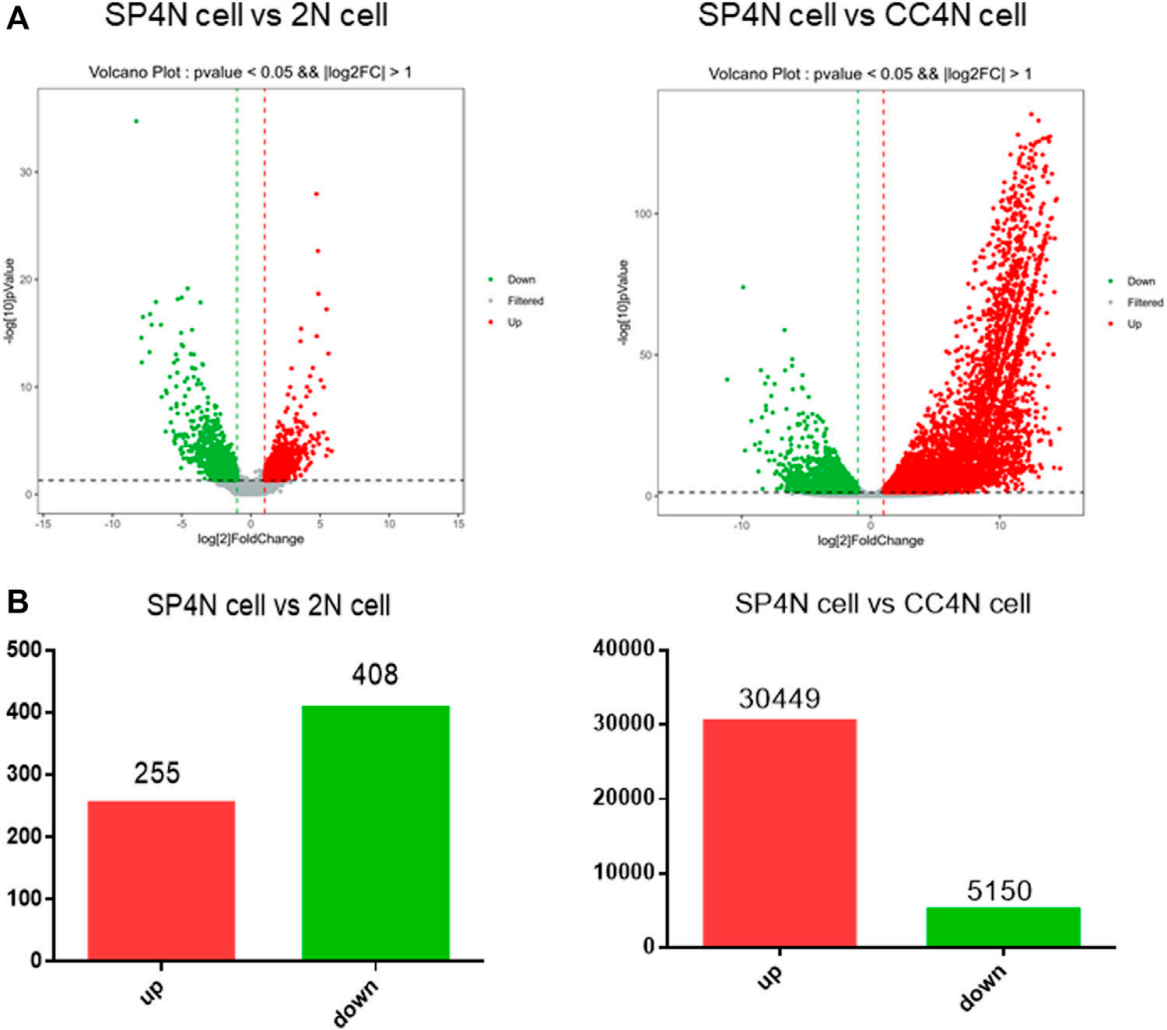
Differentially expressed genes (DEGs) between the SP600125 induced tetraploid cells (SP4N cells) from *C. auratus* × *C. carpio L.* (CC4N cells) and the diploid fish cells (2N cells). **(A)** presents the distribution of DEGs in SP4N and 2N **(left lane)**, SP4N and CC4N **(right lane)**. **(B)** presents the up- or down-regulated DEGs in the three types of cells. Different expression levels of cell cycle genes in three types of cells.

The transcriptome data of SP4N cells were compared with those of the 2N cells or the CC4N cells, collected from the NCBI SRA database. By comparison between the SP4N cells and the 2N cells, 663 differentially expressed genes (DEGs) were obtained. Among these genes, 255 (38.46%) were significantly up-regulated and 408 (61.54%) significantly down-regulated (Figure 4A). Comparing the SP4N cells with the CC4N cells, a total of 35,599 differentially expressed genes were obtained, among which 30,449 (85.53%) genes were significantly up-regulated and 5,150 (14.47%) genes were significantly down-regulated (Figure 4B). These results showed that the transcription difference between the SP4N cells and the 2N cells was smaller than that between the SP4N cells and the CC4N cells.

According to the GO and KEGG database, the DEGs of SP4N cells and 2N cells were significantly different in the cell adhesion and migration (*alcam*, *tn*, *jami*), immunity (*nik, stat1*, *cd2*), calcium ion regulation (*ncx*, *cacna1a*, *cacna2d3*), energy metabolism (*egr2*, *hk2*, *adora1*) (Supplementary Table S3). While the DEGs of SP4N cells and CC4N cells were among the tumor related genes (*cldn, fn1, twist*), transcription factors (*eif2s2, eif4e*), cycle regulation related genes (*cdc25b, cdc20*), and energy metabolism (*uqcrc2, pfka, ndufs6*) (Supplementary Table S4).

**FIGURE 5:**
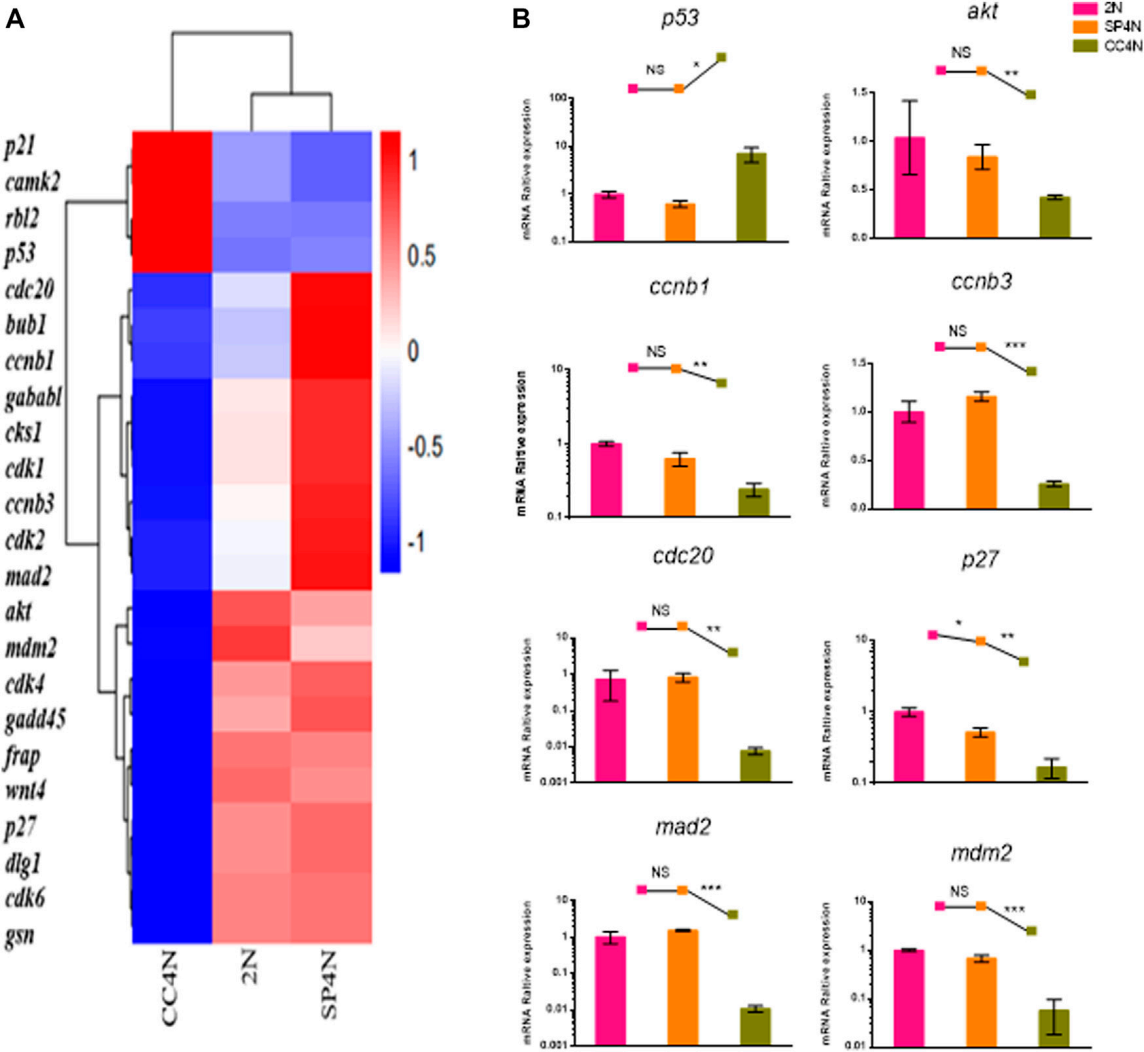
Expression levels of cell cycle regulation genes detected by mRNA-seq and qRT-PCR in the SP600125 induced tetraploid cells (SP4N cells), in the tetraploid cells from *C. auratus* × *C. carpio L.* (CC4N cells) and in the diploid fish cells (2N cells). **(A)** Heatmap of the expression distribution of cell cycle regulation genes as detected by mRNA-seq. **(B)** The expression levels of eight genes detected by qPCR. For each sample, experiments were repeated at least 3 times. “NS” stands for no significant difference, *p ≤ 0.05, **p ≤ 0.01 and ***p ≤ 0.001 represent the different significance levels of the conducted *t*-test, respectively. Analysis of mitochondria-related energy metabolism genes.

The transcriptome data demonstrated that the DEGs were involved in cell cycle regulation pathways in the SP4N and CC4N cells, but not in the SP4N and 2N cells (Figure 5A, Supplementary Figure S2 andTables S3, S4). Eight genes of cell cycle pathway were chosen to be further determined by qRT-PCR. As shown in Figure 5B, in the SP4N cells, the expression levels of these eight genes were similar to those in the 2N cells, but significantly different from those in the CC4N cells.

**FIGURE 6:**
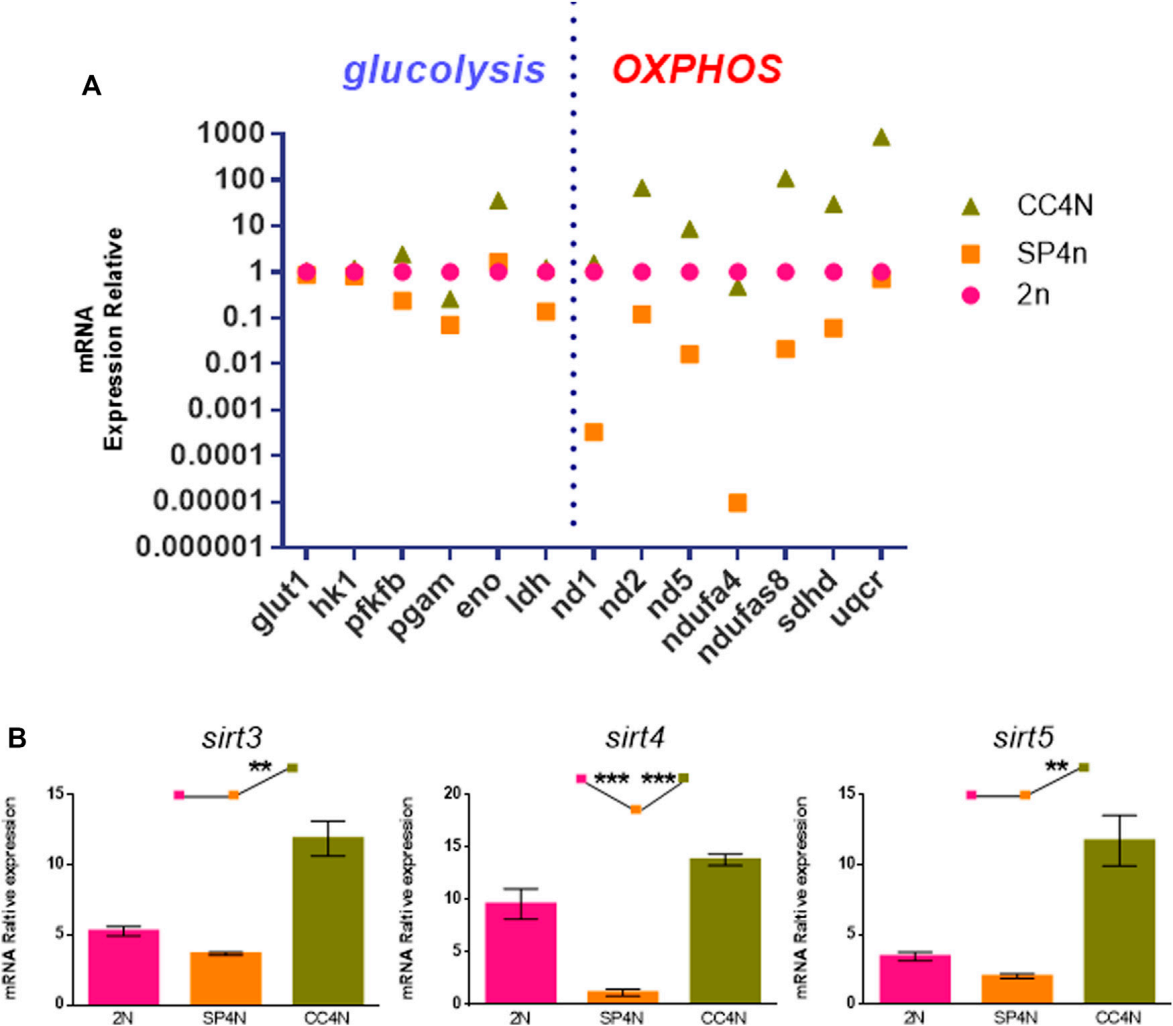
Transcriptional levels related to energy metabolism were detected in the SP600125 induced tetraploid cells (SP4N cells), the tetraploid cells from *C. auratus* × *C. carpio L.* (CC4N cells) and the diploid fish cells (2N cells). **(A)** Comparison of transcription levels of glycolysis and oxidative phosphorylation (OXPHOS) related genes in the three types of cells. **(B)** The transcriptional expression levels of *sirt* genes detected by qRT-PCR. For each sample, experiments were repeated at least 3 times.

As shown in Supplementary Table S3, S4, many energy metabolism-related genes were annotated to KEGG related pathways from transcriptome data among the SP4N cells, 2N cells and CC4N cells. We selected six glycolytic genes (*glut1*, *hk1*, *pfkfb*, *pgam*, *eno*, *ldh*) and seven oxidative phosphorylation genes (*nd1*, *nd2*, *nd5*, *ndufa4*, *ndufs8*, *sdhd*, *uqcr*) for further detection by qRT-PCR. As shown in Figure 6A, comparing with the 2N cells, most of the genes in the SP4N cells related to glycolysis and mitochondrial oxidative phosphorylation pathway were down-regulated. The transcription levels of glycolysis and mitochondrial oxidative phosphorylation related genes in the two tetraploid fish cells were significantly different where the expression level in the CC4N cells was higher than the SP4N cells. Except for *pgam* and *ndufa4*, which were slightly lower than the 2N cells, the expression levels of other detected genes in the CC4N cells were higher than the 2N cells and SP4n cells (Figure 6A).

Sirtuin family belongs to the third type histone deacetylase, especially Sirt3, Sirt4 and Sirt5, localized in mitochondria and closely associated with metabolism and oxidative respiration (Haigis et al., 2006; Jin et al., 2009; Nakagawa et al., 2009). The transcription levels of *sirt3*, *sirt4* and *sirt5* genes in the SP4N cells were the lowest among the 3 cells (shown as SP4N < 2N < CC4N) (Figure 6B).

## Discussion

In this study, it was found that the SP600125-induced tetraploid cells (SP4N cells) have many common characteristics of tetraploid, such as larger nucleus volume, four chromosome groups, and 4C nuclear DNA contents. We also proved that the SP4N cells had a normal chromosome behavior in cell proliferation. Moreover, the expression trend of the genes associated with cell cycle regulation in the SP4N cells was similar to the 2N cells, which is closely related to the stability of the SP4N cell line in cell proliferation. These results further strengthened that the SP600125 cyclic treatment is an efficient chemical induction approach to polyploid generation (Zhou et al., 2016).

Tetraploid is an organism with four chromosome groups in somatic cells. It has been shown that the tetraploid cells had chromatin instability owing to the difficulties in dividing genomes with double number of centrosomes and chromosomes (Cowell 1980; Mayer and Aguilera, 1990; Fujiwara et al., 2005; Margolis 2005; Storchova et al., 2006; Coward and Harding 2014). In general, an increasing nuclear DNA content has important effects on gene expression in cells (Hancock, Martin, and Lelchuk 1993). As shown in our previous reports, analysis of the change in the global transcriptomic profile after SP600125 treatment, a number of DEGs (4,516) were identified between the SP600125-treated cells and the 2N cells (Mo et al., 2019), and 3.52% DEGs were involved in cell cycle regulation, including the p53 signaling pathway genes and spindle assembly checkpoint genes (Xu et al., 2021). It suggested that the SP600125-induced polyploidization occurred accompanied by chromosomal abnormality, which is closely related to cell cycle regulation (Xu et al., 2021). In this study, by making comparison among the SP4N cells, the CC4N cells and the 2N cells, it was revealed that the transcription difference between the SP4N cells and the CC4N cells was more significant than that between the SP4N cells and the 2N cells. Especially, the expression trend of the genes associated with cell cycle regulation in the SP4N cells was different to that in the CC4N cells. The differential molecular regulation mechanism of autotetraploid and allotetraploid in cell proliferation remains to be further investigated.

Furthermore, cytological observation has shown that the number of mitochondria in the 2N cells and SP4N cells was more abundant than that in the CC4N cells, and had giant mitochondria in the SP4N cells. However, there was no obvious difference in terms of the levels of ATP contents among the three types of cells. According to the GO and KEGG database, we found that the abundant DEGs in the SP4N cells, 2N cells and CC4N cells were involved in the energy metabolism. Quantitative analysis further confirmed that in the SP4N cells, the expression levels of many mitochondrial function genes, such as glycolysis and mitochondrial oxidative phosphorylation pathway, were the lowest, compared with the 2N cells and the CC4N cells. In the CC4N cells, the expression levels were the highest. It seems that enhanced expression levels of energy metabolism pathways might ensure the energy demand of the CC4N cells.

## Data Availability

The datasets presented in this study can be found in online repositories. The names of the repository/repositories and accession number(s) can be found below: https://www.ncbi.nlm.nih.gov/, SRR9964682 and SRR9964683.
