# Supplementary material for: Characteristics of SP600125 Induced Tetraploid Cells in Comparison With Diploid and Tetraploid Cells of Fish
Source: Front Genet. 2021 Dec 6;12:781007. doi: 10.3389/fgene.2021.781007 (PMC8685524; doi:10.3389/fgene.2021.781007)
Supplement: Supplementary file 1 [file DataSheet1.PDF]

Supplementary Figures

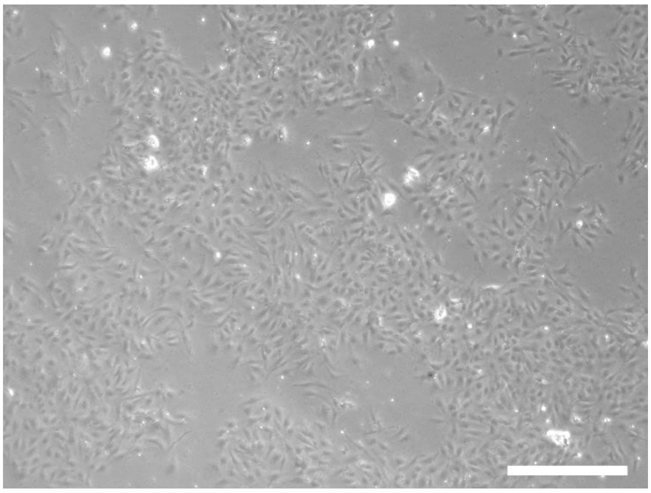

Figure S1. Morphology of the SP600125 induced tetraploid cells (SP4N cells) from 38th passage. Scale bar represents 500 μm.

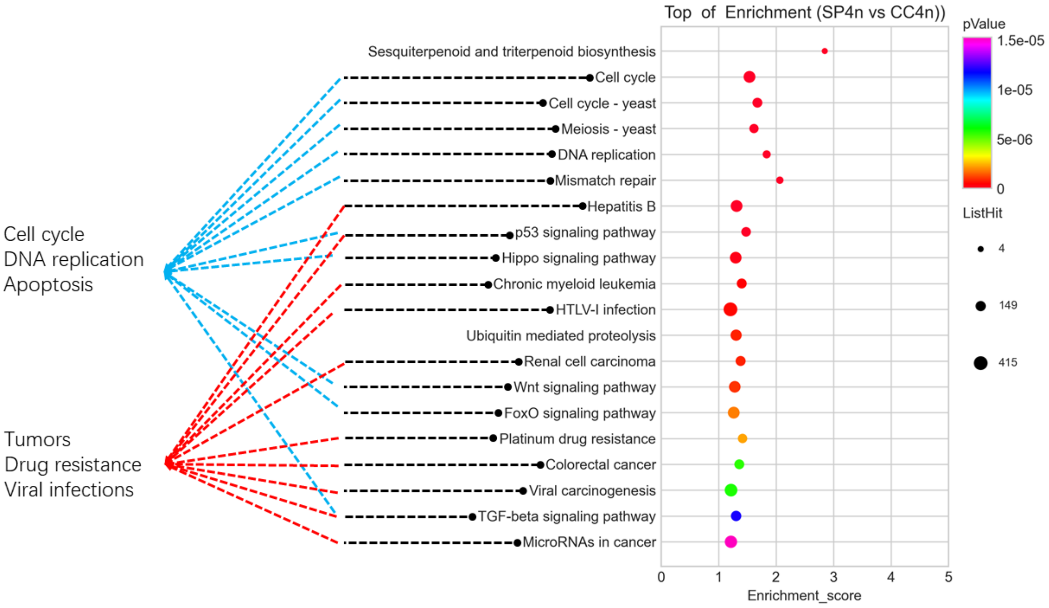

Figure S2. Differentially expressed genes (DEGs) between the SP600125 induced tetraploid cells (SP4N cells) and tetraploid cells from *C. auratus* × *C. carpio* L. (CC4N cells).

Supplementary Tables

Table S1 List of the gene name Acronyms

Gene	Note	Gene	Note
abca1	ATP-binding cassette, sub-family A (ABC1), member 1	*mcl1*	myeloid leukemia cell differntiation protein MCL-1
abcb1	ATP-binding cassette, sub-family B (MDR/TAP), member 4	*mcm2*	DNA replication licensing factor MCM2
abcc10	ATP-binding cassette, sub-family C (CFTR/MRP), member 10	*mcm3*	DNA replication licensing factor MCM3
abcc2	ATP-binding cassette, sub-family C (CFTR/MRP), member 2	*mdm2*	E3 ubiquitin-protein ligase Mdm2
actn1_4	actinin alpha 1/4	*mhc1*	MHC class I alpha chain, partial
adora1	adenosine A1 receptor	*mmp9*	matrix metalloproteinase-9 (gelatinase B)
akt	AKT2 kinase	*msh6*	DNA mismatch repair protein MSH6
alcam	Activated leukocyte cell adhesion molecule A	*myc*	Myc proto-oncogene protein
aldh1	Aldehyde dehydrogenase, mitochondrial	*mylk*	myosin light chain kinase family, member 4
alox15b	Arachidonate 15-lipoxygenase B	*ncx*	Na+/Ca2+ exchanger
angpt1	Angiopoietin 1	*nd1*	NADH dehydrogenase subunit 1
atf3	activating transcription factor 3	*nd2*	NADH dehydrogenase subunit 2
atf4	cyclic AMP-dependent transcription factor ATF-4	*nd5*	NADH dehydrogenase subunit 5
bax	apoptosis regulator BAX	*ndufa4*	NADH dehydrogenase [ubiquinone] 1 alpha subcomplex subunit 4
bcl2	apoptosis regulator Bcl-2	*ndufs8*	NADH dehydrogenase ubiquinone Fe-S 8
birc5	baculoviral IAP repeat-containing protein 5	*nik*	NF-kappa-beta-inducing kinase
bmp6	bone morphogenetic protein 6	*notch*	notch homolog 5
bub1	mitotic checkpoint serine/threonine-protein kinase BUB1 isoform X1	*npffr1*	neuropeptide FF receptor 1
cacna1a	Full=Voltage-dependent N-type calcium channel subunit alpha-1B	*nrxn*	Neurexin-2-beta
cacna2d	voltage-dependent calcium channel subunit alpha-2/delta-3 isoform X1	*ntng2*	netrin G2
camk2	calcium/calmodulin-dependent protein kinase type II subunit gamma isoform	*oxtr*	oxytocin receptor like
casp3	caspase 3	*p21*	Novel protein Fragment
ccl20	c-C motif chemokine 20-like	*p27*	Cyclin-dependent kinase inhibitor p27
ccnb1	G2/mitotic-specific cyclin-B1	*p53*	Cellular tumor antigen p53
ccnb3	G2/mitotic-specific cyclin-B3	*par1*	Proteinase-activated receptor 1
ccnd1	cyclin D1	*pfkfb*	6-phosphofructo-2-kinase/fructose-2
ccnd2	cyclin D2	*pfkfb 6*	6-phosphofructo-2-kinase/fructose-2,6-biphosphatase 2
ccng1	cyclin G1	*pgam*	cell division cycle 25 homolog d
ccr9	C-C chemokine receptor type 9	*pik3c*	phosphoinositide-3 kinase,catalytic subunit delta
cd2	T-cell surface antigen CD2-like	*pim1*	proto-oncogene serine/threonine-protein kinase Pim-1
cd90	Thy-1 cell surface antigen	*plcg2*	phosphatidylinositol phospholipase C, gamma-2
cdc20	cell division cycle protein 20 homolog	*pln*	Cardiac phospholamban
cdc42	cell division control protein 42	*pola2*	DNA polymerase alpha subunit B
cdc45	cell division control protein 45	*pold2*	DNA polymerase delta subunit 2
cdk1	Cyclin-dependent kinase 1	*pold4*	DNA polymerase delta subunit 4
cdk2	cyclin-dependent kinase 2	*pole1*	DNA polymerase epsilon subunit 1
cdk4	cyclin-dependent kinase 4	*prf1*	Perforin-1
cdk6	Cyclin-dependent kinase 6	*pri2*	DNA primase large subunit
chmp2b	chromatin modifying protein 2B	*prlhr*	brain prolactin-releasing peptide receptor-like protein 2
cks1	CDC28 protein kinase 2	*ptgs2*	prostaglandin G/H synthase 2 precursor
cldn	claudin 19	*pthr2*	parathyroid hormone 2 receptor
cntfr	ciliary neurotrophic factor receptor	*pttg*	securin
col4a	Collagen alpha-2(IV) chain	*rapgef1*	Rap guanine nucleotide exchange factor 1
cpkc	protein kinase C, alpha	*rasal2*	RAS protein activator-like 2
decr1	2,4-dienoyl CoA reductase 1	*rbl2*	retinoblastoma-like protein 2 isoform X1
dlg1	disks large homolog 1 isoform X2	*reck*	reversion-inducing-cysteine-rich protein with kazal motifs
drd4	dopamine receptor D4b	*rfa2*	replication factor A2
egr2	early growth response protein 2b	*rfc3_5*	replication factor C subunit 3/5
eno	enolase 1	*rhoa*	Ras homolog gene family, member A
epac	Rap guanine nucleotide exchange factor (GEF) 3	*sdhd*	succinate dehydrogenase
exo1	exonuclease 1	*sh3glb*	SH3-domain GRB2-like endophilin B1
fgfr3	fibroblast growth factor receptor 3	*sirt1*	NAD-dependent ADP-ribosyltransferase sirtuin-1
fn1	fibronectin variant 3, partial	*sirt2*	NAD-dependent ADP-ribosyltransferase sirtuin-2
fos	proto-oncogene protein c-fos	*sirt3*	NAD-dependent ADP-ribosyltransferase sirtuin-3

frap	mechanistic target of rapamycin	*sirt4*	NAD-dependent ADP-ribosyltransferase sirtuin-4
fzd9_10	frizzled homolog 9	*sirt5*	NAD-dependent ADP-ribosyltransferase sirtuin-5
gababl	probable G-protein coupled receptor 156-like isoform X1	*sirt6*	NAD-dependent ADP-ribosyltransferase sirtuin-6
gabbr	G protein-coupled receptor 51	*sirt7*	NAD-dependent ADP-ribosyltransferase sirtuin-7
gad	Transposon Ty3-G Gag-Pol polyprotein	*skp2*	S-phase kinase-associated protein 2
gadd45	growth arrest and DNA-damage-inducible, alpha, b	*slc2a1*	MFS transporter, SP family, solute carrier family 2 , member 1
glsa	glutaminase	*stag1_2*	cohesin complex subunit SA-1/2
glut1	glucose transporter X	*stat1*	signal transducer and activator of transcription 1b
grpr	gastrin-releasing peptide receptor	*stmn1*	stathmin
gsn	scinderin like a	*taar*	trace amine-associated receptor 10d
gst	glutathione S-transferase	*tgfb1*	transforming growth factor beta-1
h4	histone H4	*tgfb2*	transforming growth factor beta-2
hdac7	histone deacetylase 7	*tgfb3*	transforming growth factor beta-3
hif1a	hypoxia-inducible factor 1 alpha	*tgfbr2*	TGF-beta receptor type-2
hk	Hexokinase-2	*tn*	Tenascin-N
hk1	hexokinase 1	*top2*	DNA topoisomerase II
hspb1	Heat shock 27 kDa protein	*twist*	twist homolog 3
hvem	Tumor necrosis factor receptor superfamily member 5	*ube3a*	ubiquitin-protein ligase E3 A
id2	DNA-binding protein inhibitor ID2	*uqcr*	cytochrome b-c1 complex subunit 10-like
igfbp3	insulin-like growth factor binding protein 3	*usp7*	ubiquitin carboxyl-terminal hydrolase 7
irs2	insulin receptor substrate 2	*vdac3*	voltage-dependent anion channel protein 3
jam1	Junctional adhesion molecule 1	*vegfr1*	Kinase insert domain receptor like
jun	transcription factor AP-1	*vps37*	vacuolar protein sorting 37 homolog D (S. cerevisiae)
kif23	kinesin family member 23	*wnt4*	Protein Wnt-4a Precursor
ldh	L-lactate dehydrogenase A chain	*ywhab_q_*	14-3-3 protein beta/theta/zeta
mad2	spindle assembly checkpoint protein Mad2	*ywhae*	14-3-3 protein epsilon
map2k1	mitogen-activated protein kinase kinase 1	*twist*	twist
il8	interleukin 8	*nup62*	nuclear pore complex protein Nup62
oxtr	oxytocin receptor	*nup88*	nuclear pore complex protein Nup88
ngfr	nerve growth factor receptor (TNFR superfamily member 16)	*slc37a4*	solute carrier family 37, member 4
ppp1r12	protein phosphatase 1 regulatory subunit 12B	*hprt1*	hypoxanthine phosphoribosyltransferase
col5as	collagen, type V/XI/XXIV/XXVII, alpha	*tyms*	thymidylate synthase
c3	complement component 3	*eif4e*	translation initiation factor 4E
fgf	fibroblast growth factor	*pcgf4*	polycomb group RING finger protein 4
cldn	claudin	*casp3*	caspase 3
thy1	Thy-1 cell surface antigen	*hmbs*	hydroxymethylbilane synthase
fn1	fibronectin 1	*cdc20*	cell division cycle 20, cofactor of APC complex
ant	solute carrier family 25	*uqcrc2*	ubiquinol-cytochrome c reductase core subunit 2
rxfp3	relaxin family peptide receptor 3	*imp4*	U3 small nucleolar ribonucleoprotein protein IMP4
cxadr	coxsackievirus and adenovirus receptor	*g22p2*	ATP-dependent DNA helicase 2 subunit 2
chst3	chondroitin 6-sulfotransferase 3	*snrpb2*	U2 small nuclear ribonucleoprotein B"
xpnpep2	Xaa-Pro aminopeptidase 2	*ube2c*	ubiquitin-conjugating enzyme E2 C
hsd3b7	cholest-5-ene-3beta,7alpha-diol 3beta-dehydrogenase	*cdc25b*	M-phase inducer phosphatase 2
ptgds	prostaglandin-H2 D-isomerase	*acot1_2_4*	acyl-coenzyme A thioesterase 1/2/4
ppp1r3	protein phosphatase 1 regulatory subunit 3A/B/C/D/E	*calm*	calmodulin
tg	thyroglobulin	*uble1b*	ubiquitin-like 1-activating enzyme E1 B
slc12a7	solute carrier family 12 (potassium/chloride transporter), member 7	*psmb4*	20S proteasome subunit beta 7
sts	steryl-sulfatase	*pfka*	6-phosphofructokinase 1
oprd1	delta-type opioid receptor	*arfgap2_3*	ADP-ribosylation factor GTPase-activating protein 2/3
cyp2j	cytochrome P450 family 2 subfamily J	*eif2s2*	translation initiation factor 2 subunit 2
spt	serine palmitoyltransferase	*psmb3*	20S proteasome subunit beta 3
ptges3	cytosolic prostaglandin-E synthase	*rrp41*	exosome complex component RRP41
ndufs6	NADH dehydrogenase (ubiquinone) Fe-S protein 6	*nmt*	phosphoethanolamine N-methyltransferase
rp-l24	large subunit ribosomal protein L24	*rcl1*	RNA 3'-terminal phosphate cyclase-like protein

Table S2 Sequences of the primers used for qRT-PCR.

**gene name**	**sequence**	**gene name**	**sequence**
glut1-F	5'-TTTAGTCTCGGGAACACGGAAG-3'	*sirt7-F*	5'-GTTCATCCGGCTGTTTGACG-3'
glut1-R	5'-TATTCGCTCAAGGTCCACGC-3'	*sirt7-R*	5'-TGCACCACAAGCACGAGTAT-3'
pfkfb-F	5'-TCACTACTTTATTTGTAATGGTGCGAC-3'	*akt-F*	5'-AATACGACACTCGCTCCC-3'
pfkfb-R	5'-GTCAGATTTGGTGCAGTTTGTACTGT-3'	*akt-R*	5-'CACAGACACCGCACAACT-3'
pgam-F	5'-GGCTGGGATACACATTCACTCG-3'	*mdm2-F*	5'-ACACGACTGACTGAATGGGC-3'
pgam-R	5'-GCCTCATCCATCCGACTCAATC-3'	*mdm2-R*	5'-TGTCAAAGTCGCCCCAAGAA-3'
hk-F	5'-CAGCAATGCAGTCGTCTTC-3'	*ccnb3-F*	5'-TGCGGTGTGTCTGTTTCAGT-3'
hk-R	5'-AAGGCCTACTGTTTGAGGG-3'	*ccnb3-R*	5'-GATACGCTGACCAACCCCAA-3'
eno-F	5'-GTCCAGGCTTCCCAGTCATC-3'	*ccnb1-F*	5'-CCAGCAGCGAGAATCAGA-3'
eno-R	5'-CCCAGCCGTTATATCAGCCC-3'	*ccnb1-R*	5'-AGCCACAACCTCCACCTT-3'
ldh-F	5'-GTGACGCAAGTACGGAATG-3'	*p53-F*	5'-GGGAAAGGAGGTCGGTGAAG-3'
ldh-R	5'-TGTGTCTGTGTGCGCTCTAT-3'	*p53-R*	5'-GTTTCGCTGCCACAGTTCAG-3'
adprh-F	5'-AAGACTGCGTATGGGACCTG-3'	*cdc20-F*	5'-GCTGGTAGGCTTGGGTTTCT-3'
adprh-R	5'-GGCCCGTTATCAGAGGAGTG-3'	*cdc20-R*	5'-TGTGTGGTCACGGTTTTGCT-3'
nd1-F	5'-CCACCTAGATAGAAAGCG-3'	*mad2-F*	5'-TGGAGACTAATGAGGTGCTG-3'
nd1-R	5'-GTTGAGTTCGTTAGTTGCC-3'	*mad2-R*	5'-CAAGAAGGTGACTGTGGC-3'
nd2-F	5'-GGATGTTGGCGTATTCGGCT-3'	*cacna2d3-F*	5'-ACGGCCAAAGTCCTGTCATC-3'
nd2-R	5'-CTAATTCCCGCCTGGCCTTT-3'	*cacna2d3-R*	5'-CTAATGGGTCAAGGCAGGGG-3'
nd5-F	5'-CAACCGGGTAGGAGACATCG-3'	*col4a-F*	5'-TTGGCATGGCTCGTTCTGAT-3'
nd5-R	5'-GGCTGATTTTCCCGTTGCTG-3'	*col4a-R*	5'-TTCATCGAGTGTAACGGGGC-3'
ndufa4-F	5'-CACTGAAGAACCCTGACTG-3'	*gabbr2-F*	5'-CAGTTTACCCGCCCTCTGTT-3'
ndufa4-R	5'-CTGCTGTCTGATGATGTG-3'	*gabbr2-R*	5'-GATACTGGGGCGGACCTTTC-3'
ndufs8-F	5'-CCATTGTACAGACTCAAG-3'	*hk2-F*	5'-TCTAGGACGACCAAGCAT-3'
ndufs8-R	5'-CTTGTGCTGAGGTAATAAG-3'	*hk2-R*	5'-TCCAAAAAGCTGCAAGGTG-3'
sdhd-F	5'-CAGACCTTTGGCCGTACAGA-3'	*igfbp3-F*	5'-ATGGGGAAAGAGCACACGAG-3'
sdhd-R	5'-GAACTACGCTCAGGATCCGC-3'	*igfbp3-R*	5'-TGTGCAATCAACGGGTCACT-3'
uqcr-F	5'-GGTGTTGATGGCTGAATGTCC-3'	*ncx-F*	5'-GGAGAAGTTTTGCGTGCGAT-3'
uqcr-R	5'-GTCTCCATTGCCAGAACGTG-3'	*ncx-R*	5'-CACAGGCTGACAGATTGGA-3'
sirt1-F	5'-AAGAACAAGCCCTCGTCCAG-3'	*prf-F*	5'-GCCAATCGTCGTCCCATTT-3'
sirt1-R	5'-TGGTGAGGATCAGCCGTTTC-3'	*prf-R*	5'-GGGCTCAAAATCTGTGGGG-3'
sirt2-F	5'-CTTCCGCCTTTCGACACTCA-3'	*vps37-F*	5'-CTCGGACTGTTCTTCGGCAT-3'
sirt2-R	5'-TGCGCAGGAAATCCATCT-3'	*vps37-R*	5'-TCACCTTCAAAACGGCAAG-3'
sirt3-F	5'-TGCTCAGGATGTACACGCA-3'	*enc1-F*	5'-TCCGTGATGCTTGTGCCGA3'
sirt3-R	5'-CCCTTGTAATCCCTTCGGCA-3'	*enc1-R*	5'-TAGCGGGGAAGTTGCTGAGG3'
sirt4-F	5'-TGTTCCTGCAAGTGGCTC-3'	*cytb-2-F*	5'-CACTACTATTCTCCATCCTCGT3'
sirt4-R	5'-CACGTAGTTCCTTGCCCAGT-3'	*cytb-2-R*	5'-AGGAACAGTGCGAAATATAGG3'
sirt5-F	5'-GCTATCATTACGGGGGCTGG-3'	*β-actin-F*	5'-CGAGAAGATGACCCAGATCA-3'
sirt5-R	5'-GCTTCAGGGGTTGCCAAATC-3'	*β-actin-R*	5'- GATCTTCATGAGGTAGTCAG-3'
sirt6-F	5'-GTGTGAGAAGTGTGGCAAGC-3'		
sirt6-R	5'-AGGCAGATCTCCGCTAGGTT-3'		

Table S3 Comparison of some differentially expressed genes between the SP4N cells and the 2N cells.

Gene	SP4N_FPKM	2N_FPKM	P_value	Gene	SP4N_FPKM	2N_FPKM	P_value
prf1	1.33	0.01	1.05E-06	*igfbp3*	0.4	11.74	6.80E-20
rasal2	11.04	3.56	3.17E-04	*pln*	11.44	42.92	5.41E-05
alox15b	0.68	0.1	2.64E-04	*cacna1a*	0.32	2.32	3.34E-07
pik3c	1.84	0.39	6.33E-04	*pthr2*	0.21	9.04	1.64E-14
tn	31.36	1.93	1.98E-04	*prlhr*	0.01	1.15	1.17E-05
cacna2d3	2.42	0.61	5.21E-05	*oxtr*	0.02	1.04	7.32E-05
ncx	13.4	0.36	2.17E-19	*ndufa4*	0.12	33.96	1.69E-17
adora1	1.59	0.32	2.13E-04	*abca1*	0.34	4.18	7.33E-05
gabbr	0.78	0.11	4.32E-05	*abcb1*	1.41	9.45	2.58E-05
ccr9	104.66	17.68	5.17E-04	*abcc2*	5.74	13.86	1.49E-04
taar	2.37	0.33	6.47E-05	*abcc10*	1.82	7.41	1.01E-05
grpr	2.62	0.43	2.95E-04	*pfkfb 6*	0.43	3.57	2.05E-06
npffr1	1.49	0.17	2.24E-06	*epac*	0.69	6.71	1.76E-04
aldh1	100.22	36.11	3.54E-04	*gad*	0.17	1.97	7.94E-05
decr1	29.99	12.02	7.17E-04	*egr2*	8.21	36.14	1.46E-04
ptgs2	2.01	0.09	7.04E-04	*col4a*	0.12	4.25	8.76E-14
hk	13.84	5.82	5.55E-04	*cntfr*	50.12	158.12	1.73E-04
vps37	7.35	2.3	5.53E-05	*chmp2b*	0.26	1.4	2.65E-04
drd4	1.91	0.08	4.59E-04	*cldn*	0.26	3.5	1.84E-05
fzd9_10	8.03	0.49	2.02E-07	*nik*	0.08	1.07	1.35E-06
notch	2.91	0.59	2.63E-06	*hspb1*	1.87	34.13	9.47E-14
hvem	0	1.83	9.87E-05	*vegfr1*	0.04	0.6	7.30E-04
atf3	30.46	93.96	3.65E-05	*ccl20*	0	23.93	2.68E-15
par1	0	0.8	1.04E-06	*stat1*	0.07	0.69	7.27E-05
angpt1	0.03	0.58	3.07E-04	*cd2*	0	0.79	3.96E-04
sh3glb	17.27	46.22	2.25E-05	*ntng2*	0.05	2.6	1.89E-08
fn1	1.94	80.69	2.93E-13	*nrxn*	0.25	1.98	8.99E-07
alcam	0.16	1.81	3.20E-04	*cd90*	0.56	21.52	9.29E-16
jam1	0.26	1.12	7.31E-04	*mhc1*	0.05	17.83	1.25E-18
twist	0.29	2.04	6.27E-05	*mylk*	0.11	2.18	9.03E-13

Table S4 Comparison of some differentially expressed genes between the SP4N cells and the CC4N cells.

Gene	JL4N_FPKM	SP4N_FPKM	P_value	Gene	JL4N_FPKM	SP4N_FPKM	P_value
twist	27.1	0.03	5.58E-42	*nup62*	5.7	6.97	4.16E-02
il8	459.46	0.89	7.46E-17	*nup88*	4.73	5.81	1.52E-02
oxtr	15.98	0.06	2.43E-27	*slc37a4*	3.97	4.9	8.18E-03
ngfr	2.93	0.02	1.93E-09	*hprt1*	15.53	19.08	6.01E-03
ppp1r12b	8.05	0.05	1.35E-19	*tyms*	10.38	12.65	4.12E-02
col5as	91.92	0.18	2.74E-45	*eif4e*	7.83	9.73	1.53E-02
c3	7.67	0.06	3.40E-31	*pcgf4*	3.81	4.76	1.81E-02
fgf	5.51	0.07	2.08E-13	*casp3*	24.96	31.08	1.75E-03
cldn	11.92	0.05	1.23E-03	*hmbs*	6.78	8.48	6.06E-03
thy1	103.04	0.56	2.16E-07	*cdc20*	15.75	19.34	2.61E-02
fn1	103.65	0.6	1.62E-59	*uqcrc2*	30.6	37.62	1.29E-02
ant	4.61	0.08	1.06E-06	*imp4*	10.48	13.16	8.31E-03
rxfp3	1.25	0.04	1.43E-03	*g22p2*	3.71	4.64	3.35E-02
cxadr	1.28	0.04	2.29E-03	*snrpb2*	12.84	16	1.96E-02
chst3	2.36	0.06	1.23E-05	*ube2c*	26.71	33.32	1.84E-02
xpnpep2	1	0.02	3.36E-03	*cdc25b*	3.75	4.67	3.98E-02
hsd3b7	12.61	0.1	1.33E-20	*acot1_2_4*	2.08	2.63	3.43E-02
ptgds	99.51	1.17	2.23E-04	*calm*	10.33	13.09	3.85E-02
ppp1r3	2.53	0.07	7.63E-05	*uble1b*	6.32	8	5.28E-03
tg	211.48	1.86	7.12E-47	*psmb4*	70.1	88.91	8.89E-04
slc12a7	1.72	0.03	1.75E-04	*pfka*	19.91	25.05	2.19E-03
sts	1.53	0.04	1.87E-04	*arfgap2_3*	4.73	5.89	2.71E-02
oprd1	2.05	0.05	4.31E-06	*eif2s2*	74.5	93.69	2.34E-03
cyp2j	4.58	0.06	1.72E-11	*psmb3*	58.88	74.9	8.40E-04
spt	1.19	0.04	4.88E-04	*rrp41*	6.78	8.57	2.19E-02
ptges3	31.72	38.53	6.77E-03	*nmt*	10.17	12.83	8.83E-03
ndufs6	50.97	61.99	1.02E-02	*rcl1*	5.1	6.51	1.43E-02
rp-l24	14.41	17.65	1.51E-02				
